# Clinical Microbial Contamination of Multidose Brimonidine: A Prospective Comparison of Benzalkonium Chloride and Purite® Preservatives

**DOI:** 10.1155/joph/2867565

**Published:** 2026-05-25

**Authors:** Serap Karaca, Omer Faruk Yilmaz, Abdurrahman Sarmis, Muhammed Ali Mutlu, Zahide Busra Sahin, Sabire Pelin Kaya, Halit Oguz

**Affiliations:** ^1^ Department of Ophthalmology, Goztepe Prof. Dr. Suleyman Yalcin City Hospital, Istanbul, Turkey; ^2^ Department of Medical Microbiology, Goztepe Prof. Dr. Suleyman Yalcin City Hospital, Istanbul, Turkey; ^3^ Department of Medical Microbiology, Fatih Sultan Mehmet Training and Research Hospital, Istanbul, Turkey, fsmhastanesi.gov.tr; ^4^ Department of Ophthalmology, Istanbul Medeniyet University Faculty of Medicine, Istanbul, Turkey, medeniyet.edu.tr

**Keywords:** benzalkonium chloride, brimonidine, contamination, multidose ophthalmic preparations, Purite®

## Abstract

**Objective:**

To compare microbial contamination between multidose brimonidine eye drops preserved with benzalkonium chloride (BAK‐MDB) and multidose brimonidine eye drops preserved with Purite® (PUR‐MDB) using a comparative approach on the same patient under real‐life conditions.

**Materials and Methods:**

In this prospective study, 40 glaucoma patients used BAK‐MDB and PUR‐MDB for three weeks each. From each bottle, the cap and first and second drops were cultured on sheep blood and chocolate agar. Paired comparisons were performed using the exact McNemar test. Associations between patient‐related factors and contamination were evaluated using Fisher’s exact test and exploratory Firth‐penalized logistic regression.

**Results:**

The contamination rates on the caps were similar between PUR‐MDB and BAK‐MDB on sheep blood agar (12.5% vs. 12.5%) and chocolate agar (10% vs. 12.5%) (exact McNemar test, all *p* > 0.05). The contamination rates of the first and second drops were lower than that of the cap, and no statistically significant difference was found between the drops (exact McNemar test, all *p* > 0.05). In univariate analysis, no difference was observed between contamination and patient‐related factors (all *p* > 0.05). In exploratory multivariate analysis, a higher education level was independently associated with a significant reduction in contamination risk (OR: 0.09; 95% CI: 0.001–0.84; *p* = 0.031).

**Conclusion:**

In our study, no statistically significant difference was observed between BAK‐MDB and PUR‐MDB in terms of microbial contamination in caps, first and second drops. The exploratory association between education level and contamination risk will need to be confirmed in larger, adequately powered prospective studies.

## 1. Introduction

Ocular surface infections that can develop due to eye drop contamination are a significant clinical problem in ophthalmology practice [1]. In 2023, the FDA recalled several eye drops from the drug market after contamination was detected in these products. Contamination risks may increase due to administration failures such as touching the dropper tip with the hand, the dropper tip touching the eyelid, eyelashes, or eye surface, or the cap remaining prolonged open. The use of preservatives is recommended to prevent contamination in multidose bottles [2, 3]. Preservatives have an important role in preventing microbial growth in multidose drops by protecting the sterility of the drug and extending its storage duration [4].

Approximately 70% of eye drops have contained benzalkonium chloride (BAK) as a preservative since the 1950s. BAK is a preservative with high antimicrobial activity [5]. However, it also exhibits significant toxicity [6]. Due to this high toxicity, less toxic alternative preservatives or preservative‐free eye drops are increasingly being used. Among the alternative preservatives are stabilized oxychloro complex 0.005% (Purite®), polyquaternium (Polyquad), sodium perborate, and the ionic‐buffered preservative SofZia. Studies support that these alternative preservatives are less toxic [2, 7]. Although the toxicity profile of BAK has been extensively studied, clinical data comparing its contamination resistance with Purite®, which is promoted as a safer alternative preservative, are insufficient.

To our knowledge, there are no prospective clinical studies comparing microbial contamination of multidose brimonidine eye drops preserving with benzalkonium chloride (BAK‐MDB) and multidose brimonidine eye drops preserving with Purite® (PUR‐MDB) during routine clinical use. There are two equivalent multidose formulations containing brimonidine available in our country. Therefore, in this study, BAK‐MDB and PUR‐MDB were prospectively compared for microbial contamination under routine clinical conditions by administering both formulations sequentially to the same patients.

## 2. Materials and Methods

This prospective study was conducted between August 1, 2024, and December 5, 2024, at the glaucoma unit of Istanbul Goztepe Prof. Dr. Suleyman Yalcin City Hospital. The study included a total of 80 multidose eye drops used by 40 glaucoma patients (23 women and 17 men). This was a crossover clinical study in which each patient used both formulations sequentially, minimizing interindividual variability.

Patients diagnosed with glaucoma and using only brimonidine‐containing drops in both eyes were included in this study. Patients had been using a monotherapy antiglaucoma drop for at least 1 year. Patients with eyelid disorders, topical or systemic infections, patients receiving immunosuppressive therapy, topical or systemic antibiotics or corticosteroids, and patients using artificial tears were excluded from the study.

Patients were randomized to start with either BAK‐MDB or PUR‐MDB using a simple randomization procedure. Patients were instructed to instill one drop of each preservative‐containing solution into each eye twice daily (12 h apart) for three weeks. After using BAK‐MDB for 3 weeks, the patient was prescribed PUR‐MDB to use for the next 3 weeks; after using PUR‐MDB for 3 weeks, and the patient was prescribed BAK‐MDB drops to use for the next 3 weeks. A new bottle of drops was opened each time. Microbiological analyses were performed by researchers who had no knowledge of the preservative.

In this study, Gloger eye drops containing BAK‐MDB (Biem Pharmaceuticals Ltd., Turkey) were compared with Alphagan P eye drops containing PUR‐MDB (Allergan Inc., Irvine, CA, USA). Patients were asked who administered the drops, their level of education, whether they administered the drops while sitting or lying down, whether the tip of the bottle touched the eyelid or skin, whether they paid attention to hand hygiene, and which drop was more difficult to use.

Patient‐level contamination was defined as the presence of microbial growth in at least one sample, including the cap, first drop, second drop, or bottle contents (any contamination). The outcome measurements included the presence of microbial contamination on the multidose bottle caps, the first and second drops. Additionally, the relationships between contamination and patient‐related factors were evaluated.

### 2.1. Bacteriological Culture

BAK‐MDB and PUR‐MDB multidose eye drops were evaluated for contamination using sterile swab sticks (Microcult, Ankara, Turkey). The caps and the first and second drops from the bottles were inoculated onto 5% sheep blood agar and chocolate agar (BioMerieux, France). Swabs were carefully applied to the inner surfaces of the caps to avoid contamination. Each plate was incubated at 37°C for 48 h. Any colony that grew on the agar was collected individually using a sterile loop. Identification of the isolates was performed on the same day using the Vitek MS system (BioMerieux, France).

The outside of contaminated bottles was disinfected for at least 1 minute using 96% ethanol on a sterile gauze pad (Amasya Sugar Factory Ltd., Turkey). The remaining contents inside the bottle was then removed using a 30‐gauge insulin needle (Vera Global, London, United Kingdom). Sterile conjunctival swabs were collected from both eyes of patients with pathogenic microorganism growth and directly inoculated onto chocolate agar and 5% sheep blood agar at the bedside. The agar plates were incubated at 37°C for 48 h in a 5% CO_2_ incubator (Ildam, Turkey). The bacterial colonies grown were identified using the Vitek MS system (BioMerieux, France).

### 2.2. Statistical Analysis

Comparisons between PUR‐MDB and BAK‐MDB were performed using the exact McNemar test for paired data. Effect sizes were reported as paired odds ratios (OR) with 95% confidence intervals (CI) calculated using the exact conditional method. Analyses were considered exploratory, and no adjustments were made for multiple comparisons. Associations between patient‐related characteristics and patient‐level microbial contamination were assessed using Fisher’s exact test. An exploratory multivariate analysis was performed using Firth‐penalized logistic regression with prespecified variables to reduce small sample bias and address potential data separation. All statistical tests were two‐sided, and a *p*‐value < 0.05 was considered statistically significant. Statistical analyses were performed using R statistical software (R Foundation for Statistical Computing, Vienna, Austria).

## 3. Results

Cap contamination was detected in 5/40 cases (12.5%) in the PUR‐MDB group and in 5/40 cases (12.5%) in the BAK‐MDB group on sheep blood agar. On chocolate agar, it was observed in 4/40 cases (10%) in the PUR‐MDB group and in 5/40 cases (12.5%) in the BAK‐MDB group; no statistically significant difference was found between the groups (exact McNemar test, all *p* > 0.05) (Table 1, Table 2).

**TABLE 1:** Microbial growth on bottle caps and bottle contents in multidose brimonidine: benzalkonium chloride (BAK‐MDB) versus Purite® (PUR‐MDB).

Patient number	Contamination of caps of PUR‐MDB (*n* = 40)		Contamination of caps of BAK‐MDB (*n* = 40)		Bottle content culture
	Sheep blood agar	Chocolate agar	Sheep blood agar	Chocolate agar	
3	*Staphylococcus epidermidis*	*Staphylococcus epidermidis*	—	—	—
4	—	—	*Staphylococcus epidermidis*	*Staphylococcus epidermidis*	—
6	*Candida famata*	*Candida famata*	—	—	—
16	*Staphylococcus aureus*	*Staphylococcus aureus*	—	—	—
19	*Staphylococcus epidermidis*	*Staphylococcus epidermidis*	—	—	—
20	—	—	*Enterobacter aerogenes*	*Enterobacter aerogenes*	*Enterobacter aerogenes*
24	—	—	*Priestia megaterium*	*Priestia megaterium*	—
27	—	—	*Staphylococcus saprophyticus, Achromobacter denitrificans*	*Staphylococcus saprophyticus, Achromobacter denitrificans*	—
38^∗^	*Staphylococcus epidermidis*	—	*Pantoea agglomerans*	*Pantoea agglomerans*	—
Total	**5/40 (12.5%)**	**4/40 (10%)**	**5/40 (12.5%)**	**5/40 (12.5%)**	**1/40 (2.5%)**

*Note:* BAK‐MDB: benzalkonium chloride‐preserved multidose brimonidine; PUR‐MDB: Purite®‐preserved multidose brimonidine. The number of patients with microbial growth was provided to indicate contamination; identifiable patient information was not included. Bold values indicate the total number of contaminations and the corresponding percentages for each culture medium.

^∗^Additionally, pathogenic microbial growth was detected only in the conjunctival culture of patient number 38.

**TABLE 2:** Paired analyses of site‐specific microbial contamination between multidose brimonidine: benzalkonium chloride (BAK‐MDB) versus Purite® (PUR‐MDB).

Outcome	PUR‐MDB contaminated *n*/*N* (%)	BAK‐MDB contaminated *n*/*N* (%)	Discordant pairs (BAK/PUR)	OR (95% CI)	*p* value^∗^
Caps sheep blood agar	5/40 (12.5%)	5/40 (12.5%)	5/3	1.67 (0.32–8.62)	0.73
Caps chocolate agar	4/40 (10%)	5/40 (12.5%)	6/3	2.00 (0.43–12.36)	0.51
First drop sheep blood agar	3/40 (7.5%)	2/40 (5.0%)	1/2	0.50 (0.01–9.60)	1.00
First drop chocolate agar	1/40 (2.5%)	2/40 (5.0%)	2/1	2.00 (0.10–117.99)	1.00
Second drop sheep blood agar	3/40 (7.5%)	2/40 (5.0%)	1/2	0.50 (0.01–9.60)	1.00
Second drop chocolate agar	1/40 (2.5%)	2/40 (5.0%)	2/1	2.00 (0.10–117.99)	1.00

*Note:* OR, paired odds ratio between BAK‐MDB and PUR‐MDB. The 95% confidence intervals (CI) were calculated using the exact conditional method. Discordant pairs indicate patients contaminated with only one preservative‐containing formulation.

^∗^Exact McNemar test.

For the first drops, microbial contamination was observed in 3/40 cases (7.5%) in the PUR‐MDB group and in 2/40 cases (5%) in the BAK‐MDB group on sheep blood agar. On chocolate agar, it was detected in 1/40 (2.5%) cases in the PUR‐MDB group and 2/40 (5%) cases in the BAK‐MDB group, with no significant difference between the groups (exact McNemar test, all *p* > 0.05) (Table 3, Table 2).

**TABLE 3:** Microbial contamination in the first drop: multidose brimonidine preserved with benzalkonium chloride (BAK‐MDB) versus Purite® (PUR‐MDB).

Patient number	Contamination of first drop of PUR‐MDB (*n* = 40)		Contamination of first drop of BAK‐MDB (*n* = 40)	
	Sheep blood agar	Chocolate agar	Sheep blood agar	Chocolate agar
6	*Candida famata*	—	—	—
16	*Staphylococcus aureus*	*Staphylococcus aureus*	—	—
20	—	—	*Enterobacter aerogenes*	*Enterobacter aerogenes*
38	*Staphylococcus epidermidis*	—	*Pantoea agglomerans*	*Pantoea agglomerans*
Total	**3/40 (7.5%)**	**1/40 (2.5%)**	**2/40 (5%)**	**2/40 (5%)**

*Note:* BAK‐MDB: benzalkonium chloride‐preserved multidose brimonidine; PUR‐MDB: Purite®‐preserved multidose brimonidine. The number of patients with microbial growth was provided to indicate contamination; identifiable patient information was not included. Bold values indicate the total number of contaminations and the corresponding percentages for each culture medium.

Second drop contamination was detected in 3/40 (7.5%) cases in the PUR‐MDB group and 2/40 (5%) cases in the BAK‐MDB group on sheep blood agar; in 1/40 (2.5%) cases in the PUR‐MDB group and 2/40 (5%) cases in the BAK‐MDB group on chocolate agar. No significant difference was observed between the preservatives (exact McNemar test, all *p* > 0.05) (Table 4, Table 2).

**TABLE 4:** Microbial contamination in the second drop: multidose brimonidine preserved with benzalkonium chloride (BAK‐MDB) versus Purite® (PUR‐MDB).

Patient number	Contamination of second drop of PUR‐MDB (*n* = 40)		Contamination of second drop of BAK‐MDB (*n* = 40)	
	Sheep blood agar	Chocolate agar	Sheep blood agar	Chocolate agar
6	*Candida famata*	—	—	—
16	*Staphylococcus aureus*	*Staphylococcus aureus*	—	—
20	—	—	*Enterobacter aerogenes*	*Enterobacter aerogenes*
38	*Staphylococcus epidermidis*	—	*Pantoea agglomerans*	*Pantoea agglomerans*
Total	**3/40 (7.5%)**	**1/40 (2.5%)**	**2/40 (5%)**	**2/40 (5%)**

*Note:* BAK‐MDB: benzalkonium chloride‐preserved multidose brimonidine; PUR‐MDB: Purite®‐preserved multidose brimonidine. The number of patients with microbial growth was provided to indicate contamination; identifiable patient information was not included. Bold values indicate the total number of contaminations and the corresponding percentages for each culture medium.

**FIGURE 1:**
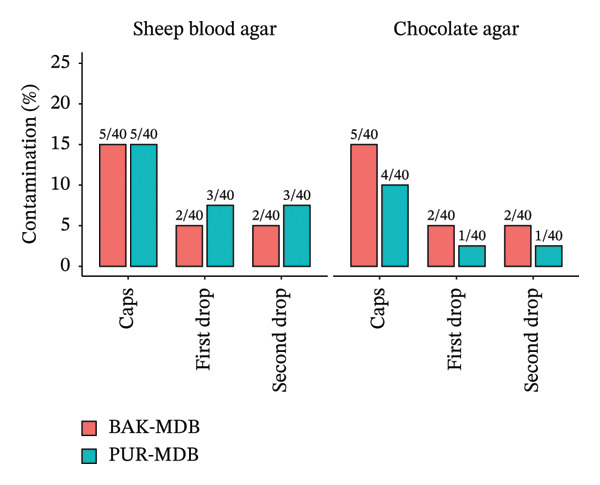
Comparison of microbial contamination in the caps, first drops, and second drops of multidose brimonidine eye drops preservative with benzalkonium chloride (BAK‐MDB) and Purite® (PUR‐MDB) on sheep blood agar and chocolate agar.

In paired analyses, no statistically significant differences were observed between PUR‐MDB and BAK‐MDB in terms of cap, first, and second drop contamination on sheep blood agar and chocolate agar (exact McNemar test, all *p* > 0.05) (Table 2). These findings are illustrated in Figure 1.

In univariate analysis, no statistically significant relationship was found between microbial contamination at the patient level and education level, gender, age, instillation position, hand washing habits, contact with the dropper, and instillation by another person (all *p* > 0.05) (Table 5).

**TABLE 5:** Univariate analysis of patient‐related factors associated with patient‐level microbial contamination.

Characteristic	Any contamination absent, *n* (%)	Any contamination present, *n* (%)	OR (95% Cl)	*p* value^∗^
Education level				
Primary school	18 (58.1)	6 (66.7)		
High school	4 (12.9)	3 (33.3)	0.45 (0.09–2.37)	0.38
University	9 (29.0)	0 (0.0)	6.68 (0.34–131.56)	0.15
Gender				
Female	19 (61.3)	4 (44.4)		
Male	12 (38.7)	5 (55.6)	0.52 (0.13–2.20)	0.45
Age				
< 60	9 (29.0)	2 (22.2)		
≥ 60	22 (71.0)	7 (77.8)	0.79 (0.16–3.99)	1.00
Who dripped the eye drops				
Self‐administered	26 (83.9)	9 (100.0)		
Administered by another person	5 (16.1)	0 (0.0)	3.94 (0.20–78.30)	0.57
Hand‐washing				
Performed	24 (77.4)	7 (77.8)		
Not performed	7 (22.6)	2 (22.2)	0.92 (0.18–4.78)	1.00
Instillation position				
Sitting	18 (58.1)	8 (88.9)		
Lying down	13 (41.9)	1 (11.1)	4.14 (0.64–26.89)	0.12
Contact				
Present	4 (12.9)	2 (22.2)		
Absent	27 (87.1)	7 (77.8)	0.49 (0.09–2.81)	0.60

*Note:* OR indicates the odds ratio for patient‐level microbial contamination. Reference categories are shown in the first row of each variable. Odds ratios and 95% confidence intervals were calculated using exact methods. *p* values were calculated using Fisher’s exact test. Values are presented as *n* (%).

^∗^Fisher’s exact test.

In exploratory multivariate analysis using Firth‐penalized logistic regression, a higher education level was independently associated with a significantly reduced risk of contamination (OR: 0.09; 95% CI: 0.001–0.84; *p* = 0.031), but gender did not demonstrate a significant association with the risk of contamination (*p* = 0.573) (Table 6).

**TABLE 6:** Multivariable analysis of patient‐related factors associated with patient‐level microbial contamination.

Variable	Odds ratio (95% CI)	*p* value
Education level		
Primary/high school (reference)	1.00	—
Higher education	**0.09 (0.001–0.84)**	**0.031**
Gender		
Female (reference)	1.00	—
Male	1.51 (0.35–6.53)	0.573

*Note:* Odds ratios (OR) and 95% confidence intervals (CI) were estimated using Firth‐penalized logistic regression to reduce small sample bias and address potential data separation. Reference categories are indicated in the first row of each variable. *p* values are based on the penalized likelihood ratio test. Bold formatting indicates statistically significant results.

## 4. Discussion

Preservatives are known to reduce microbial contamination. In a study by Yilmaz et al. [1], which evaluated 410 topical medications from 22 different pharmaceutical companies for contamination, it was found that preservatives reduced bacterial contamination on bottle caps. In our study, we evaluated the caps, first drops, and second drops containing the same active ingredient but two different preservatives (BAK and Purite®) for microbial contamination. In our study, the contamination rates in the caps, first drops, and second drops on both agar plates were similar in the BAK‐MDB and PUR‐MDB groups, and no statistically significant difference was observed.

BAK is a cationic compound with potent bactericidal and fungicidal activity that induces an inflammatory response [8]. It is a mixture of alkyl benzyl dimethyl ammonium chloride, part of the quaternary ammonium salts class. BAK functions as a surfactant or detergent, disrupting the lipid components of cell membranes. Compared to other preservatives, it has a longer shelf life and lower production costs. The concentration of BAK used in eye drops ranges between 0.04% and 0.02% [9]. Among alternative preservatives, Purite® breaks down into water, oxygen, sodium, and chloride ions when it comes into contact with the eye. When instilled onto the ocular surface, it converts into natural tear components. It consists of 99.5% chloride, 0.5% chlorate, and trace amounts of chlorine dioxide [6]. The chlorine dioxide component is responsible for its antimicrobial activity. Purite® is known to cause the oxidation of intracellular lipids and glutathione. It has a broad‐spectrum antimicrobial effect against bacteria, viruses, and certain fungi [10].

BAK is a broad‐spectrum and effective disinfectant, but it is highly toxic [11]. The threshold concentration at which toxicity occurs is estimated to be 0.005% [4]. Umetsu et al. [12] demonstrated that even at very low concentrations, BAK disrupts cellular metabolic functions and significantly affects cell viability. It can cause damage to the cornea, conjunctiva, goblet cells, limbal stem cells, ocular surface disease, and trabecular meshwork cells [4, 5, 13, 14]. Suanno et al. [15] observed an increase in corneal fluorescein staining in mice treated with artificial tears containing 0.2% BAK and reported that corneal epitheliopathy improved after BAK discontinuation. Aydemir et al. [16] conducted a retrospective study on 83 eyes that had been using preservative‐containing antiglaucoma drops for at least 1 year. They found a significant decrease in corneal hysteresis and corneal resistance factor in formulations containing BAK. Studies have also shown that BAK disrupts the ocular microbiota and increases the number of antibiotic‐resistant bacteria [17].

Purite® has fewer side effects compared to BAK [5]. Walsh and Jones [6] demonstrated in animal studies that Purite® is less toxic to the corneal epithelium than BAK. Similarly, Eraslan and Celikay [18] divided 106 glaucoma patients into two groups based on their use of Purite® or BAK‐containing medications. They found that while corneal epithelial thickness and central corneal thickness remained unchanged in the Purite® group, these parameters decreased in the BAK group. Another study showed that Purite® has a lesser impact on corneal resistance compared to BAK. Aydemir et al. [16] also demonstrated that Purite® is less cytotoxic than BAK. In ex vivo experiments, Izzotti et al. [19] compared the DNA damage induced by BAK and Purite® in trabecular meshwork cells and found that Purite® caused less damage.

In vitro studies also showed that Purite® had a lower impact on trabecular meshwork cell viability [19]. Noecker et al. [20] conducted a study on 15 New Zealand white rabbits, exposing 30 eyes to Purite®, glaucoma medications containing different BAK concentrations, and artificial tears. After 30 days, corneal damage and conjunctival inflammation were more pronounced in the eyes treated with high‐concentration BAK‐containing glaucoma medications. Similarly, Alonso et al. [21] reported that BAK‐containing eye drops induced more inflammation than those containing Purite®. These studies highlight the importance of preferring alternative preservatives such as Purite®, Polyquad, sodium perborate, and Sofzia over BAK [19, 20].

In our study, conjunctival swabs were collected from patients whose eye drops showed pathogenic microbial growth. Conjunctival colonization was detected in only one patient. This patient had *Staphylococcus epidermidis* growth in their PUR‐MDB bottle and *Pantoea agglomerans* growth in their BAK‐MDB bottle. On sheep blood agar, the patient’s conjunctiva showed growth of *S. epidermidis*, *Corynebacterium macginleyi*, and *Staphylococcus lugdunensis*, while on chocolate agar, *S. epidermidis* and *S. lugdunensis* were identified. Since the patient had no symptomatic complaints and their ophthalmologic examination was normal, no treatment was initiated. However, the importance of personal hygiene was explained in detail.

Yilmaz et al. [1] reported higher contamination rates among individuals who were illiterate or had only completed primary school education; Davis et al. [22] demonstrated that low educational level was associated with incorrect eye drop administration techniques; Tatham et al. [23] identified educational level as an independent predictor of correct drop administration in a multivariate analysis. Zhang et al. [24] also observed lower rates of incorrect drop application among patients with higher education levels; however, this relationship was not statistically significant (*p* = 0.06). Consistent with these findings, no microbial growth was observed in the drops used by patients with university education in our study. Although the relationship between education level and contamination was not statistically significant in the univariate analysis, these results may be interpreted considering the limited sample size and discrete data structure. Importantly, in the multivariate Firth penalized logistic regression analysis, higher educational level was independently associated with a significant reduction in contamination risk. In conclusion, despite the relatively small sample size, our findings support that a higher educational level may be associated with lower contamination.

Chantra et al. [25] detected contamination in 24.06% of 295 preservative‐free eye drops. A total of 26 different pathogens were identified, with mold being the most frequently detected. The study concluded that contamination could originate from human and environmental flora, emphasizing the importance of proper usage and storage conditions for eye drops. In our study, two bacterial species and one fungal species were observed growing on chocolate and sheep blood agar on the caps of PUR‐MDB bottles. The identified Gram‐positive bacteria were *Staphylococcus aureus* and *S. epidermidis*, while the fungal species was *Candida famata*. *Candida famata* is an environmental pathogen and was detected on the cap and in the first and second drops of PUR‐MDB.

Additionally, the dropper bottles used by the patients were punctured under sterile conditions for microbiological assessment. *Enterobacter aerogenes*, a bacterium commonly found in the intestinal flora, was isolated from only one of these bottles. This same pathogen was also detected in the BAK‐MDB bottle, as well as on the cap, first, and second drop of the medication used by the same patient. These findings suggest that the patient did not adhere to proper hand hygiene and may have contaminated the dropper tip by direct contact. No microbial growth was observed in other dropper bottles.

Other studies have evaluated the toxicity of preservatives in tissues. BAK causes conjunctival inflammation, squamous metaplasia, epithelial apoptosis, and subconjunctival fibrosis. These alterations also reduce the success rate of filtration surgery if glaucoma surgery becomes necessary [2, 4, 21]. Boimer and Birt [26] demonstrated in a retrospective analysis of 128 patients who underwent trabeculectomy between 2004 and 2006 that preoperative exposure to high doses of BAK was associated with a shorter time to surgical failure. Moreover, prolonged BAK exposure was identified as a risk factor for surgical failure. The concentration and duration of BAK use have a significant effect on the viability of goblet cells. Zhang et al. [27] reported a reduction in both the number and size of conjunctival goblet cells following BAK treatment.

In addition to ocular surface toxicity, BAK has been definitively associated with complications in deeper ocular tissues [28]. Brignole‐Baudouin et al. [29] conducted a study on rabbits and histologically demonstrated the presence of BAK in the trabecular meshwork and even around the optic nerve. The accumulation of BAK along the optic nerve can lead to vision‐threatening consequences [30]. Chronic exposure to BAK has been reported to contribute to the development of glaucoma or its progression [29, 31, 32]. Studies by Rasmussen et al. [31] and Baudouin et al. [32] have shown that BAK induces structural and functional alterations in the trabecular meshwork, which may further increase the progression of the disease.

Nagstrup [33] demonstrated that BAK containing latanoprost increases the release of IL‐6 and IL‐8 from retinal ganglion cells compared to preservative‐free latanoprost. Similarly, El et al. [34] reported that prostaglandin analogs containing BAK induce greater inflammation, ocular symptoms, and conjunctival hyperemia. Goto et al. [35] identified BAK as a potent inducer of inflammation in lens epithelial cells and reported it as the most harmful preservative. The cytotoxicity of BAK increases with its concentration [36]. Reducing the number of drops administered may improve ocular tolerance. It is believed that discontinuing the use of BAK‐containing eye drops could relatively reduce adverse effects [4]. Furthermore, improvements in side effects have been reported to be time‐dependent [31].

Gandolfi et al. [37] conducted a randomized study on 371 patients comparing BAK‐containing travoprost and preservative‐free travoprost. They found no difference in the efficacy of the two formulations in lowering intraocular pressure. They concluded that avoiding BAK exposure while effectively reducing IOP would be a more preferable option. Similarly, Jaenen et al. [38] analyzed data from 9658 patients who used either preserved or preservative‐free beta‐blocker eye drops between 1997 and 2003. They found that symptoms such as pain, stinging, burning, foreign body sensation, and dry eye sensation were more prevalent in the preserved group. Patients who switched to formulations with fewer preservatives or preservative‐free drops saw a significant reduction in symptoms. These findings suggest that the use of BAK‐containing eye drops should be avoided, particularly in patients with dry eye disease.

In a study conducted by Katz [39], a 12‐month follow‐up revealed that allergic symptoms were 41% lower in the brimonidine Purite® 0.15% group compared to the brimonidine BAK 0.2% group. Additionally, patient satisfaction and comfort were higher in the Purite® group. Similarly, Oyejide et al. [40] demonstrated in rabbits that Purite®‐containing eye drops caused less ocular discomfort, ocular hyperemia, and goblet cell loss compared to BAK‐containing drops and were better tolerated. In our study, six patients reported difficulty using the PUR‐MDB drops, while 16 patients reported difficulty using the BAK‐MDB drops. The most frequently reported complaints were burning and stinging sensations. Duru and Ozsaygili [41] conducted a study on 42 eyes using either preservative‐containing or preservative‐free brimonidine drops and found no significant difference in ocular tolerance at the 1‐month follow‐up.

In our study, no significant difference was found between patients using BAK‐MDB and those using PUR‐MDB in terms of handwashing habits and bottle contact. The majority of the microorganisms identified in our study originated from the ocular and periocular skin flora. On the caps of BAK‐MDB bottles, six bacterial species were isolated on chocolate and sheep blood agar. These included the Gram‐positive *S. epidermidis*, *Staphylococcus saprophyticus*, and *Priestia megaterium*, as well as the Gram‐negative *E. aerogenes* and *P. agglomerans*. The detection of *E. aerogenes* and *P. agglomerans*, which are commonly found in the intestinal flora, suggests poor hygiene practices and inadequate handwashing. Additionally, the presence of *Priestia megaterium*, which is part of the hand flora, indicates potential contact between the dropper tip and the hand, followed by its transfer to the periocular tissues.

This study has some limitations. Although a within‐patient crossover design was used to reduce inter‐individual variability, no washout period was applied between treatment periods. In addition, since the sample size was relatively limited, the absence of a statistically significant difference should be interpreted as insufficient evidence of a lack of difference rather than equivalence between the preservatives.

In conclusion, this prospective study found that the contamination rates of the cap, first drop, and second drop of BAK‐MDB and PUR‐MDB bottles were similar. Although numerical differences were observed in some samples, these were not statistically significant. The findings suggest that factors other than the type of preservative, such as patient‐related variables and dropper usage habits, may contribute to microbial contamination. Larger scale studies are needed to confirm these findings and to evaluate the risk of contamination in routine clinical use in more detail.

## Author Contributions

Serap Karaca, Zahide Busra Sahin, and Sabire Pelin Kaya collected the data and wrote the main manuscript. Abdurrahman Sarmis, Omer Faruk Yilmaz, and Muhammed Ali Mutlu analyzed and interpreted the patient data. Abdurrahman Sarmis and Muhammed Ali Mutlu performed laboratory tests. Serap Karaca, Omer Faruk Yilmaz, and Halit Oguz designed the work and substantively revised the article.

## Funding

There is no funding for this article.

## Disclosure

All authors read and approved the final manuscript.

## Ethics Statement

Ethical approval for this study was obtained from the Istanbul Medipol University Clinical Research Ethics Committee on 04.07.2024. Decision number: 653.

## Consent

The study participants provided informed consent for the publication of their data.

## Conflicts of Interest

The authors declare no conflicts of interest.

## Data Availability

The data that support the findings of this study are available from the corresponding author upon reasonable request.
